# A Medication Adherence App for Children With Sickle Cell Disease: Qualitative Study

**DOI:** 10.2196/mhealth.8130

**Published:** 2019-06-18

**Authors:** Kristina Curtis, Anastasiya Lebedev, Elisa Aguirre, Stephan Lobitz

**Affiliations:** 1 Health Behaviour and Interventions Research Faculty of Health and Life Sciences Coventry University Coventry United Kingdom; 2 Public Health Warwickshire Warwickshire County Council Warwick United Kingdom; 3 Klinik für Pädiatrie mS, Onkologie/Hämatologie Charité-Universitätsmedizin Berlin Berlin Germany; 4 Division of Psychiatry University College London London United Kingdom; 5 Research and Development Department North East London Foundation Trust Goodmayes Hospital Essex United Kingdom; 6 Amsterdam Street Children's Hospital Cologne Germany

**Keywords:** children, adolescents, sickle cell disease, medication adherence, models, theoretical, mHealth

## Abstract

**Background:**

Young people with sickle cell disease (SCD) often demonstrate low medication adherence and low motivation for effectively self-managing their condition. The growing sophistication of mobile phones and their popularity among young people render them a promising platform for increasing medication adherence. However, so far, few apps targeting SCD have been developed from research with the target population and underpinned with theory and evidence.

**Objective:**

The aim of this study was to develop a theory-and-evidence-based medication adherence app to support children and adolescents with SCD.

**Methods:**

The Behavior Change Wheel (BCW), a theoretically based intervention development framework, along with a review of the literature, 10 interviews with children and adolescents with SCD aged between 12 and 18 years, and consultation with experts informed app development. Thematic analysis of interviews provided relevant theoretical and evidence-based components to underpin the design and development of the app.

**Results:**

Findings suggested that some patients had lapses in memory for taking their medication (capability); variation in beliefs toward the effectiveness of medication and confidence in self-managing their condition (motivation); a limited time to take medication; and barriers and enablers within the changing context of social support during the transition into adulthood (opportunity). Steps were taken to select the appropriate behavioral change components (involving behavior change techniques [BCTs] such as information on antecedents, prompts/cues; self-monitoring of the behavior; and social support) and translate them into app features designed to overcome these barriers to medication adherence.

**Conclusions:**

Patients with SCD have complex barriers to medication adherence necessitating the need for comprehensive models of behavior change to analyze the problem. Children and adolescents require an app that goes beyond simple medication reminders and takes into account the patient’s beliefs, emotions, and environmental barriers to medication adherence.

## Introduction

### Sickle Cell Disease—A Global Health Priority

Sickle cell disease (SCD) is among the most prevalent hereditary blood disorders in the world [1], leading the World Health Organization to prioritize it as a global health issue in 2006 and 2010. SCD causes red blood cells to become *sickle-shaped* which restricts the flow of blood and the transportation of oxygen [2]. Life-threatening complications include infections, acute chest syndrome, stroke, and multiorgan failure [3]. However, by far, the most frequent complications are acute vaso-occlusive events resulting in severe pain episodes. The episodes (also known as pain crises) are predominantly managed within the home environment but require hospitalization when there are complications or when the pain becomes too great [4].

In the past decade, several new medications have been developed to improve the duration and quality of patients’ lives [5], resulting in medication adherence becoming fundamental to patients’ self-management of their condition. However, systematic review evidence indicates that medication adherence is moderate among children and adolescents [5]. Poor adherence results in reduced effectiveness of medication, increased susceptibility to complications, and medication wastage [6,7].

It is paramount that health care professionals support young patients in developing autonomy and self-management skills [8]. The challenge globally is the serious shortage of health care services to provide support for patients with SCD. The rising migrant populations necessitate the need now more than ever to provide accessible services despite language, culture, finances, ethnicity, and geolocation. Consequently, this has led to growing interest in developing tailored electronic health technologies to support the day-to-day needs of patients. Research has demonstrated an increase in medication adherence among children using short message service (SMS) technology [2], whereas other research has achieved a medication adherence rate of 93% by using electronic directly observed therapy [9].

### Sickle Cell Disease Mobile Health Apps

Mobile health (mHealth) apps *offer state-of-the-art approaches to intervention design, delivery and diffusion of treatment and prevention efforts* [10]. Key behavior change techniques (BCTs) important for self-management are optimized through this medium, such as self-monitoring techniques [11], which continue to increase in sophistication [12]. So far, there is growing evidence related to the acceptability and usability of SCD apps aimed at monitoring various symptoms such as pain and fatigue [13-15] and enabling medication reminders [9,16]. They can also enhance communication with health care providers, provide general health management [14], and provide therapeutic interventions such as cognitive behavioral therapy [17,18]. A recent systematic review [19] reported the efficacy of 1 mobile app feasibility study in improving medication adherence after a 6-month follow-up [20]. However, more research is needed to evaluate the mobile app’s efficacy and effectiveness for self-managing SCD using careful methods and theoretical underpinnings [19].

A 2013 Cochrane review of asthma self-management apps [21] concluded that future app-based interventions should be underpinned with relevant theoretical frameworks to identify the impact of individual app features on patient outcomes [19]. However, a recent content analysis of 166 medication adherence apps showed that the use of evidence-based BCTs were low [22]. According to Carpenter et al, development of app features that truly implement theoretical constructs remains an undeveloped area, and most medication adherence and disease management apps fail to report any theoretical underpinnings [23], including SDC apps [21,24]. A mobile app is a cost-effective health care intervention [25,26] to support patients’ medication adherence. Therefore, this study aimed to develop a theory and evidence-based medication adherence app for children and adolescents with SCD.

### Intervention Development Framework

The Behavior Change Wheel (BCW), a theoretically based intervention development framework, was used to guide app development [27]. The BCW is coherent, grounded in a model of behavior (described in the Methods section), and inclusive of all possible intervention strategies. The research draws on a core component of the BCW: the Capability Opportunity, Motivation-Behaviour (COM-B) model that helps to identify important levers for change for the new behavior to occur. It then uses the next steps in intervention development to help bring about change in the new behavior through identifying intervention functions (IFs) and BCTs.

The BCW framework accepts that behavior can essentially derive from a combination of theoretical components within a behavioral system [27]. The research also draws on the next layers of the wheel, IFs, which are defined as expansive classifications through which an intervention can modify behavior [27]. The 9 IFs identified are as follows: *education* (increasing knowledge or comprehension), *persuasion* (evoking emotions to stimulate action), *incentivization* (an expectation of rewards for behavior), *coercion*, (expectation of punitive consequences and costs), *training* (transmitting skills), *restriction* (using rules and regulation to reduce behavior), *environmental restructuring* (modifying the physical or social environment), *modeling* (providing an exemplar of behavior for people to emulate), and *enablement* (increasing the means to carry out the behavior) [27]. IFs can be further broken down into strategies enabling behavior change labeled as BCTs, representing the observable, replicable, and active ingredients in an intervention that directly bring about behavior change [28].

## Methods

The app development process drew on the BCW for guidance on understanding the target behavior of medication adherence and how to address this behavior through the use of relevant BCTs. The following section provides details of the steps taken during this process.

### Stage 1: Understanding the Behavior

The first stage involved a number of steps to understand the behavior.

#### Step 1: Defining the Problem

Stakeholder meetings with pediatricians along with a review of the literature helped to define the problem.

#### Step 2: Selecting the Target Behavior

Stakeholder meetings helped to consider all potential self-management behaviors. However, as previously noted, the BCW recommends starting with only 1 or 2 target behaviors and gradually building on these [22,27]. Selecting the target behavior involved consultation with 2 pediatricians with expertise in SCD and e-learning and a review of the literature.

#### Step 3: Specifying the Target Behavior

The behavior was then specified for this target population in terms of the context in which the target behavior occurs.

#### Step 4: Identifying What Needs to Change

This step involved conducting empirical research using a qualitative research design guided by the COM-B model [29] and Theoretical Domains Framework (TDF [30,31]) to explore barriers and enablers to patients’ capability, opportunity, and motivation toward medication adherence. The TDF is a framework that amalgamates central theoretical constructs from a wide range of behavior change theories. It classifies 14 significant domains such as skills and emotion, which influence behavior and are possible targets for change [31]. The TDF can be demarcated into 3 core elements of human behavior: capability (C), opportunity (O), and motivation (M) [32]. The COM-B model purports that behavior (B) is a consequence of the interactions between a person’s physical and psychological capabilities (C) to utilize social and environmental opportunities (O) via automatic or reflective motivations (M) [33]. The qualitative research involved conducting 10 interviews with children and adolescents with SCD.

### Participants

Patients were recruited through Charité University Hospital, Department of Pediatric Hematology and Oncology. All patients were considered eligible if they suffered from SCD, were treated with hydroxycarbamide, owned a smartphone, and were aged between 12 and 18 years. They were invited to take part in the development of an app at the end of their routine face-to-face consultation with the pediatrician. Upon agreement, patients were then telephoned to arrange a time to conduct the interview. Information on demographics, technology use, and smartphone ownership was collected before the interviews (see Results section). Ongoing analysis was conducted across interviews until it became clear that no new codes were emerging from the data, and therefore, recruitment for new participants ceased [34].

### Procedure

Interviews took place in a private room in the hospital where parent informed consent and child informed consent were obtained where necessary, before interviews commenced. Participants were paid 30 euros for their participation in the research. A non-native German speaking female interviewer (37 years old) conducted the interviews lasting 30 min. The interviews consisted of semi-structured questions (see Table 1 for the schedule of topics) developed from a review of existing research [5,9,35-37] and structured using the COM-B and TDF to explore barriers and enablers to patients’ capability, opportunity, and motivation to self-manage their condition, with a focus on medication adherence. For example, the TDF domains of memory, attention, and decision-making processes included questions such as “What are your thoughts on how well you remember to take your medication”, and environmental context and resources included questions such as “What are your thoughts on the things in your environment that make it difficult to take your medication?” Upon permission of the participants, the interviews were audio-recorded, transcribed, and translated into English for analysis.

### Thematic Analysis of the Interview Data

Demographic data were analyzed using descriptive statistics. Transcripts were analyzed by 2 independent researchers using recognized principles for conducting thematic analysis [38]. This involved deductively coding the data for their basic meaning before mapping to the COM-B and TDF. This analysis helped to perform a behavioral analysis of the problem wherein theoretical domains were identified as targets for change [29]. In addition, the reliability of the qualitative data was further enriched by the use of an additional trained qualitative researcher who was familiar with the BCW framework and TDF, who independently coded 10% of the data to establish interrater reliability. An agreement of 11/14 TDF domains were established on discussion, and full agreement was reached. An interrater reliability of .79 is generally considered to be an acceptable rate [39].

### Stage 2: Identifying Intervention Strategies

#### Step 5: Identifying Intervention Functions

According to Michie et al, the *behavioral diagnosis* drawn from the COM-B and TDF tools for understanding the behavior represents the foundations for intervention design. Once the *profile* of COM-B and TDF domains has been identified as important *levers for change*, the next stage is to select from a range of IFs provided by the BCW framework [27].

The BCW framework provides a table mapping relevant IFs likely to bring about change in specific COM-B and TDF domains to help conduct this task. However, it was also necessary to review the BCTs that the BCW has mapped to IFs to see how they align with the TDF domains identified. Therefore, the mapping process underwent a cyclical process where BCTs were mapped back to IFs. The next step involves delineating these IFs into specific BCTs. The authors of the guide purposely used the term *functions* to indicate that BCTs can have more than 1 IF [27].

**Table 1:** Schedule of questions for interviews.

COM-B^a^ model and TDF^b^		Topic question
**Psychological capability**		
	Knowledge	What are your thoughts on how much you know about Sickle Cell Anemia (Prompt: Can you describe what it is?) What are the complications of the condition? How is the condition treated? How does the medication (specify which one) for the condition work?
	Skills	Do you know how to take your medication? What are your thoughts on any measures that you can take to prevent the condition getting worse?
	Memory, attention, and decision-making processes	What are your thoughts on how well you remember to take your medication? Do you normally set a reminder to take your medication?
	Behavioral regulation	Do you have a way of monitoring whether you have taken your medication every day? (If no, why not? If yes, how?)
	Environmental context and resources	What are your thoughts on the things in your environment that make it difficult to take your medication? (Prompt: lack of time, lack of privacy)
**Social opportunity**		
	Social influences	What are your thoughts on how much support you receive from your parents for your condition? What kind of support do you receive from your local community? What kind of support do you receive from your close friends? What kind of support do you receive from your school/teachers? Do you have any further ideas on how family and friends can support you in an app?
**Reflective motivation**		
	Social identity	—^c^
	Beliefs about capabilities	How confident do you feel about managing your condition? (Prompt: remembering to take your medication, managing your moods, managing the pain)
	Optimism	—
	Beliefs about consequences	What do you believe might happen to your body if you take your medication? (Prompts: what are your beliefs on whether medication will make your illness worse or better?) What are your thoughts around side effects? What are your thoughts around how serious your condition is? What are your thoughts on whether it takes too much time and effort to take your medication during your daily routine?
	Intention	Do you intend to take your medication every day? (Prompt: If not, why not?)
	Goals	What are your thoughts on your goals for taking medication?
**Automatic motivation**		
	Reinforcement	What would be an incentive to take your medication? We have had some ideas of how features in an app can help to incentivize children to take their medication
	Emotion	What are your thoughts on whether your moods make your physical symptoms worse or better? How could the app help you to manage your moods better? Does taking your medication cause any emotional reactions and feelings?

^a^COM-B: Capability Opportunity, Motivation-Behavior.

^b^TDF: Theoretical Domains Framework.

^c^Not applicable.

#### Step 6: Identifying Behavior Change Techniques

Mapping BCTs to intervention functions involved 2 steps: First, the BCW table for mapping IFs to relevant BCTs provided a candidate list of BCTs to use for the intervention. As previously mentioned, selecting IFs also required looking forward to ascertain which BCTs that the BCW maps to IFs aligned with the TDF domains and context of an app. Therefore, some BCTs were already selected if they were relevant to bringing about change in the TDF domain. This step also involved reviewing a systematic review of medication adherence among pediatric patients with SCD [5].

#### Step 7: Translating Findings Into App Features

Consultation with the project team in the form of a workshop (app developers, pediatricians, and behavioral scientist) helped to translate the intervention mapping results into app features. The process involved the behavioral scientist (KC) presenting key findings using the intervention mapping table to the project team. Specifically, the table helped to communicate which BCTs were required to change medication adherence behavior. This then instigated discussions on how these techniques could be operationalized in the app, leading to decisions on app features. Further meetings via Skype also involved the design company (comprised user experience experts) to help with the development of engaging app features. Table 2 below presents the sample demographics and mobile phone usage.

**Table 2:** Demographics and mobile phone information (N=10).

Demographics		Values
**Origins of parents (N=20), n (%)**		
	Lebanon	7 (35)
	Nigeria	6 (30)
	Angola	3 (15)
	Sierra Leonne	2 (10)
	The Congo	1 (5)
	Palestine	1 (5
**Gender, n (%)**		
	Female	6 (60)
	Male	4 (40)
Age (years), mean (range)		14 (11-17)
German citizenship, n (%)		8 (80)
**Living status, n (%)**		
	Not living alone	9 (90)
Number of people living in household, average (range)		4 (3-6)
**Parents’ employment status, n (%)**		
	Mothers unemployed (n=9)	5 (56)
	Mothers employed full time (n=9)	4 (44)
	Fathers employed full time (n=7)	4 (57)
	Fathers employed part time (n=7)	2 (29)
	Fathers self-employed (n=7)	1 (14)
Smartphone ownership and access to the internet, n (%)		10 (100)
**Frequency of mobile phone use, n (%)**		
	Daily	9 (90)
	2-3 times a week	1 (10)

## Results

### Step 1: Defining the Problem

The problem was defined by the stakeholder group as too many children and adolescents with SCD not successfully self-managing their condition.

### Step 2: Selecting the Target Behavior

Pediatricians reported that medication nonadherence was a serious problem among their patients. This was also bolstered with systematic review evidence indicating medication adherence is low among young people, resulting in detrimental effects to their health [5-7]. Therefore, supporting children and adolescents with their medication adherence was identified as the target behavior.

### Step 3: Specifying the Target Behavior

The global term, *medication adherence*, incorporates initiating the prescription, actual dosing in relation to the prescription, and persisting with treatment. Adherence relates simply to the behavior itself—using treatment at the right time, for the right period, in the right quantity, and in the right manner [40]. Table 3 below specifies the target behavior by detailing who needs perform the behavior, when, where, how and with whom.

### Step 4: Identifying What Needs to Change

The behavioral analysis (shown in Table 4) revealed barriers and enablers in all 3 COM-B domains and the following 10 TDF domains involving the following: limited knowledge related to the disease itself and how the mediation works (knowledge); Forgetting to take medication and set reminders (memory, attention, and decision processes); lack of external monitoring and reminders (behavioral regulation); limited confidence related to medication adherence (beliefs about capabilities); limited importance of regular medication adherence, priority for other influences on health such as religion and negative consequences on taste and social life (beliefs about consequences); perceived limited support required during the transition to adulthood (social identity); intrinsic goals such as excelling at sports provided motivation to medication adherence (goals); emotional responses to taking medication and managing pain (emotion); perceived limited time to take medication, being outside of the home environment and other health professionals (environmental context and resources); and an over reliance on parents to remember to take medication and importance of peer support (social influences). The subthemes taken forward for intervention development are highlighted in italics in Table 4 below.

### Steps 5, 6, and 7: Intervention Mapping Table

The intervention mapping table shown below (Table 5) shows the mapping of determinants, intervention strategies, and potential app features. This is where the results from the COM-B and TDF analysis were mapped onto 15 BCTs as guided by the BCW.

**Table 3:** Specifying the target behavior.

Target behavior	Medication adherence
Who needs to perform the target behavior?	Children and young people with sickle cell disease
When do they need to perform the behavior?	Every day (at a time suitable for their schedules)
Where do they need to perform the behavior?	At home and outside of the home
How often do they need to perform the behavior?	Once a day
With whom do they need to perform the behavior?	By themselves (or parents)

**Table 4:** Behavioral analysis of the influences on patients’ medication adherence. Subthemes in italics were taken forward for intervention development.

COM-B^a^ model, TDF^b^, and subtheme			Quote
**Psychological capability**			
	**Knowledge**		
		Perceived good knowledge of self-management of condition	I don’t have the same stamina, as other teenagers of my age. I have to dress warmly in the winter, or else I’ll have pain. I’m more susceptible when I do sports, I’m not supposed to overexert myself, I can get injured quickly. My hips are inflamed because of overexertion during sports [P10].
		Perceived good knowledge of task/environment “what to do”	You have to go see Dr. Lobitz every 3 months, once a year an annual check-up, when they run all the tests. I also have to take penicillin every morning and every evening, and Siklos every evening, and Ibuprofen when I’m in pain [P10].
		*Limited knowledge in relation to how the medication works—scientific rationale*	*Don’t know [P2].* *It’s a medication for not having pain. If you don’t drink enough, this pill helps [P3].*
		*Limited disease knowledge*	*Not so much. There are these white blood cells that get into your bones [P4].*
	**Skills**		
		Good knowledge and skills in relation to how to take the medication	1 ½ film-coated tablets every day. You can take them in the morning or in the evening [P6].
		Good knowledge and skills in relation to prevention	I’m more susceptible when I do sports, I’m not supposed to overexert myself, I can get injured quickly. My hips are inflamed because of overexertion during sports [P10]. ...dressing warmly, if you’ve been swimming, put a towel on right away [P2]
	**Memory, attention, and decision-making processes**		
		Medication taking is part of a routine	It’s just something I do, take a pill before I go to bed. It’s become my routine. If I’m on a school trip or spending the night at a friend’s house, I also bring it along and take it [P9].
		*Forgetting to take medication*	*Sometimes I forget, pretty often, actually. My mom helps me remember, my dad and my sister too [P2].* *Very poorly. I need this app. I always forget [P6].*
		*Forgetting to set external reminders*	*Yes, I set the alarm clock on my cell phone for nine, for example. It helps, but sometimes I forget to set the alarm clock [P2].*
	**Behavioral regulation**		
		*Lack of external monitoring device for medication intake*	*No – I haven’t ever considered it [P5].*
**Reflective motivation**			
	**Intentions**		
		High intentions and adherence to morning and evening medication intake	It goes without saying. In the morning I wake up, I take my penicillin and I go out. In the evenings it’s also like that, I just take them. If I’m spending the night at a friend’s house, I take the medications first and then I go to my friend’s house [P10].
	**Beliefs about capabilities**		
		*Self-confidence: high and moderate confidence in relation to how well they can self-manage the condition*	*Very confident. I’m old enough to know what I have to do [P2].* *50/50. Taking my medication is the only problem I have [P6].*
	**Beliefs about consequences**		
		*High and low importance and relevance to the perceived consequences of nonadherence to treatment*	*Skipping it one day is not a biggie, but if you don’t take it for several months, you’ll get weaker, you’ll feel worse, you’ll have no more strength or energy. The pain will probably also get worse [P10]* *If I don’t take my medication, it doesn’t make any difference [P6].*
		*Religion has a greater importance compared with medication*	*Then my mother told me, God will not let you die at the age of 40 because you haven’t taken your medication [P6].*
		*Thoughts on medication intake negative consequences (physical)*	*XXX is uncomfortable to swallow. It doesn’t have any taste, but it starts dissolving in your mouth, that’s a disgusting feeling [P10].*
		*Thoughts on medication intake negative consequences (social)*	*It bothers me when I’m at a friend’s house, and we’re talking, and then I have to break up the conversation to say, I have to take my pill now. That’s annoying [P8].*
	**Social identity**		
		*Personal identity*	*I am older and I can manage [P10].*
	**Goals**		
		*Clear goals driving intrinsic motivation*	*I have a goal, to be able to be the best I can be at sports. That’s my motivation [P10].*
**Automatic motivation**			
	**Reinforcement**		
		Physical consequences of not taking medication long term	...if you skip it more often, you can get sick [P5].
		Remembering to take medication is easier when it becomes a habit	I always remember, it’s a question of habit [P3].
		The home environment	I put the pills somewhere where I’ll see them right away, someplace where I’ll definitely see them this evening. That works well [P9].
	**Emotions**		
		Variation in beliefs toward the connection between emotions and pain	There’s no connection. When I have pain, I just take medication; I always feel the same [P10]. Thinking of good things helps when you’re in pain [P7]. When something bad happens, pain gets worse [P2].
		*Negative emotion toward medication taking*	*It sometimes makes me sad, because I don’t want to be taking medicine, but I have to [P7].*
**Social opportunity**			
	**Social influences**		
		*Peer support during unwell periods*	*My friends know about my condition. They send me messages on WhatsApp when I’m sick. When I write to my class on WhatsApp that I’m sick and not coming to school, they all write to me and tell me to get better. This makes me happy [P1].*
		*Practical parental support for taking medication*	*If I’m watching TV, my mother comes and tells me I should take my medication [P7].* *They help me. They prepare my medication for me so that I can take it quickly and get on doing other things in my daily routine [P3].*
		*Emotional parental support for pain crises*	*But home when I’m with my mom, I can be like, “Aaa mama I have this stomach ache!” [P6].*
		*(Minimal) parental support only important support explicitly perceived as needed (older children)*	*My mother accompanies me sometimes to my appointments with Dr XXX. Gradually she’s been helping me less and less, I’m old enough already. We’ve both decided that I can go to those appointments on my own [P10].*
		*Peer support for medication adherence and management of condition*	*If I’m staying at a friend’s house late, I take my medication with me and my friends tell me when to take it [P7]; If we’re in PE, they tell me to sit down and tell the teacher if I’m not feeling well. When we go swimming, they come out of the water with me and go with me to fetch something to eat and drink, because I’m not supposed to stay in the water for too long. They ask me whether I’ve taken my medication [P5].*
**Physical opportunity**			
	**Environmental context and resources**		
		*Limited time to take medication*	*When I have to get dressed quickly and go to school, my school is far away, I have to do everything quickly. I don’t have enough time and then I forget [P2].*
		*Being outside of the home environment*	*When I come home from the street, I don’t think about it [P6]; If I’m out and about or if I have to take it at a different time [P2]; When I’m at a friend’s house, every once in a while I forget to take my medication [P5].*
		*Other health professionals*	*When [my regular] doctors aren’t there, the others don’t know how to treat me [P7].*

^a^COM-B: Capability Opportunity, Motivation-Behavior.

^b^TDF: Theoretical Domains Framework.

**Table 5:** Final intervention mapping table.

COM-B^a^ model, TDF^b^ and subtheme				Intervention functions		Behavior change techniques		App features
**Psychological capability**								
	**Knowledge**							
		Perceived disconnection between thoughts and moods and effects on their condition	Education		Information about antecedents, self-monitoring of behavior		Quiz, mood tracker	
		Limited knowledge in relation to how the medication works—scientific rationale	Education		Information about health consequences		Quiz related to biological causes of sickle cell disease and effects of medication	
	**MADMP^**c**^**							
		Forgetting to take medication	Environmental restructuring		Prompts/cues		Medication reminders	
	**Behavioral regulation**							
		Perceived behavioral control: Not using external monitoring device for medication intake	Environmental restructuring		Self-monitoring of the behavior, feedback on the behavior, social support (practical)		Medication diary. Record how many tablets they have taken each day and when they have missed one. Buddy messenger—A chosen close friend can also monitor medication adherence and send a reminder them to take medication	
**Reflective motivation**								
	**Beliefs about consequences**							
		Little importance and relevance of the perceived consequences to medication adherence (religion)	Education		Information about health consequences		Quiz, persuasive messages (in-app notifications). Explain that not taking medication can result in a pain crises and serious complications	
		Negative consequences on medication intake (physical)	Enablement		Problem solving		Quiz, tips	
		Thoughts on medication intake negative consequences (social)	Enablement		Problem solving		Quiz, tips	
		Perceived limited time to take medication	Education enablement		Information about health consequences, problem solving		Quiz, tips	
		Self-confidence in remembering to take medication	Environmental restructuring enablement		Problem solving, prompts/cues		Medication reminders, tips	
	**Social Identity**							
		Personal identity, transition to adulthood	Education, modelling		Information about health consequences, problem solving, social comparison, modeling		Quiz, tips, patients’ stories on how they are coping with their condition. Users can view each other’s avatars to see how many points they have for medication adherence and appointments	
		Group identity (interest in how other patients are coping)	Modeling		Social comparison, modeling		Patients stories on how they are coping with their condition	
	**Goals**							
		Clear goals driving intrinsic motivation	Persuasion		Goal setting (outcome)		Encourage them to set their own goals for taking their medication (prompt: so they can take part in sports, hang out with friends)	
**Automatic motivation**								
	**Emotion**							
		Connection between stress and pain	Enablement		Reduce negative emotions, Practice habit formation		Comical anecdotes (jokes) and videos to distract from the pain, relaxation videos, and audios	
		Negative emotion toward medication taking (reliance and interference with social activities)	Enablement, modeling		Reduce negative emotions, Social support, Problem solving, Action planning		Comical anecdotes (jokes), other patient stories on how they manage their medication adherence, messages to friends, encourage them to make a plan of how to take medication when they are socializing	
**Social Opportunity**								
	**Social influences**							
		Peer support	Environmental restructuring, enablement		Social support (practical and emotional)		Tips. Advise to call or text a friend when they need to distract from the pain	
**Physical opportunity**								
	**Environmental context and resources**							
		The home environment	Environmental restructuring		Add objects to the environment		Tips. Advise to keep their medication on show in a place in their home they will see it	
		Outside of the home environment	Environmental restructuring, enablement		Prompts/cues, Action planning, Behavioral practice		Medication reminders, encourage a plan to take medication when going out socially, instruct the person to practice taking medication in a social context	
		Other health professionals	Environmental restructuring, enablement		Adding objects to the environment		Health record	

^a^COM-B: Capability Opportunity, Motivation-Behavior.

^b^TDF: Theoretical Domains Framework.

^c^Memory, attention, and decision-making processes.

### Overall Concept of MyMate&Me

**Figure 1:**
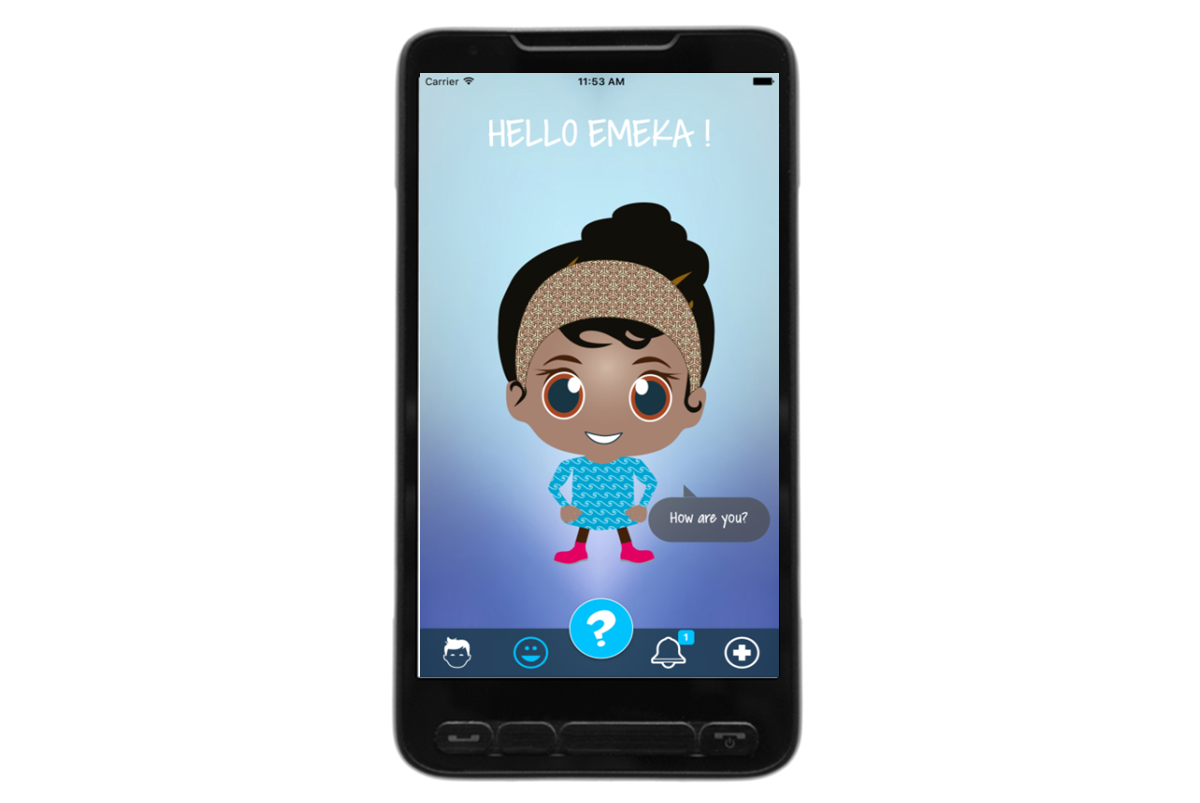
Avatar.

**Figure 2:**
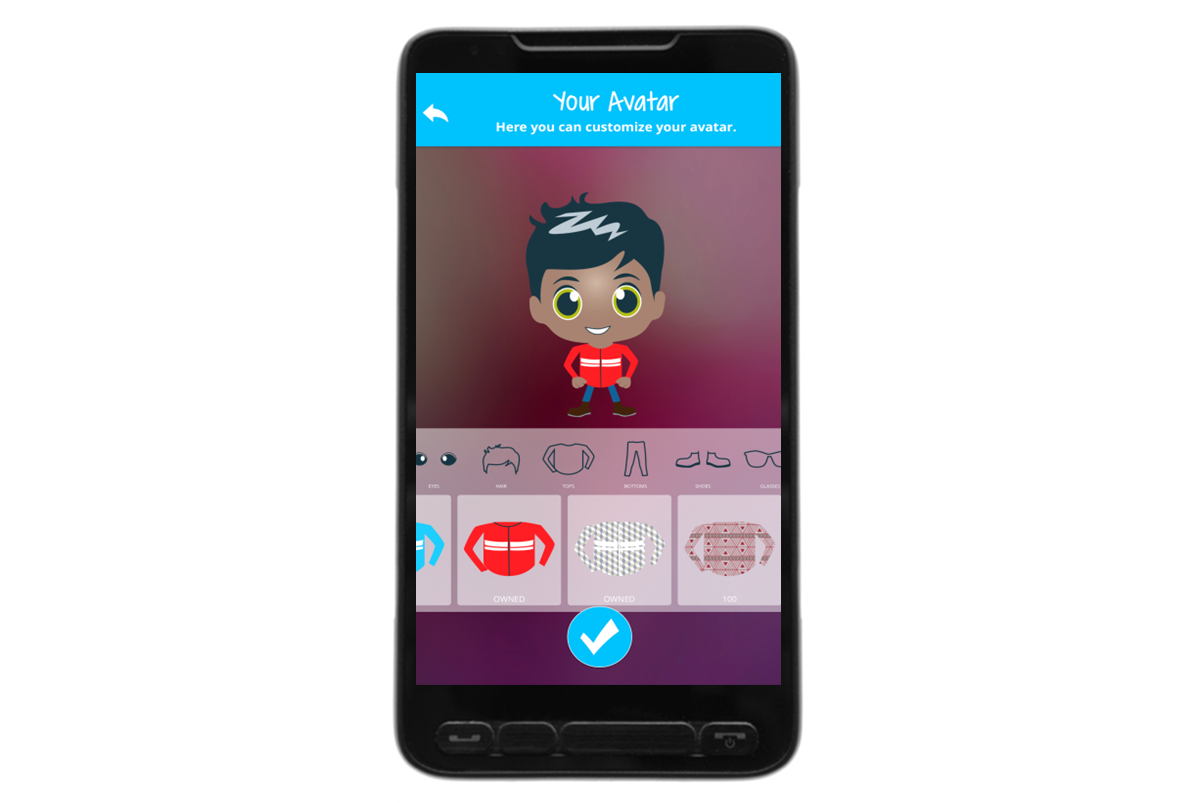
Avatar with clothes options.

**Figure 3:**
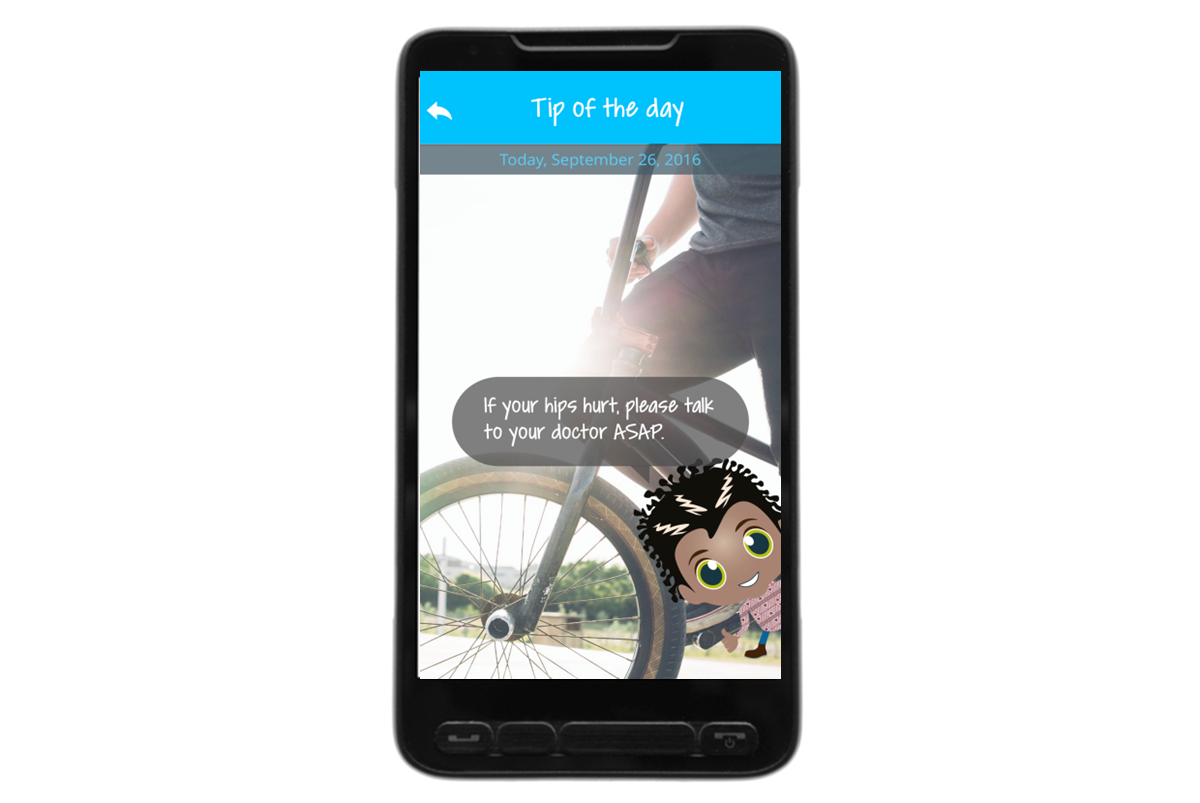
Tip of the Day.

**Figure 4:**
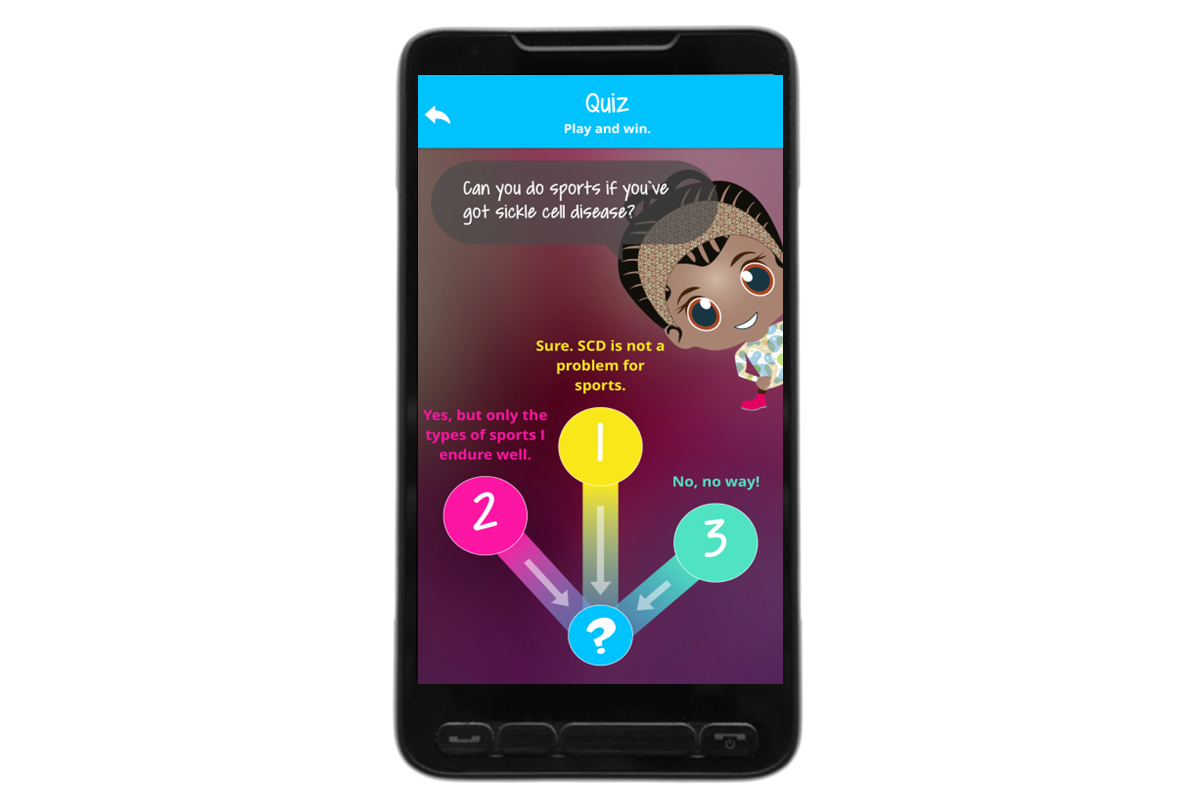
Daily Quiz.

**Figure 5:**
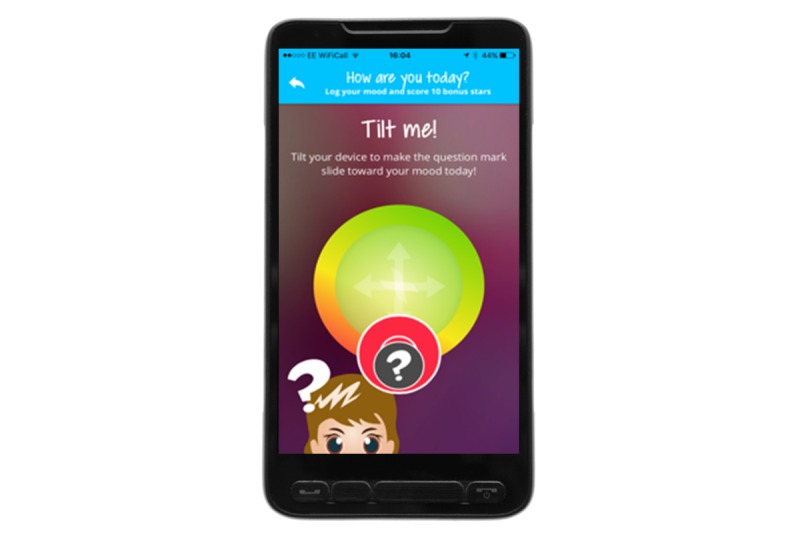
Mood Tracker.

**Figure 6:**
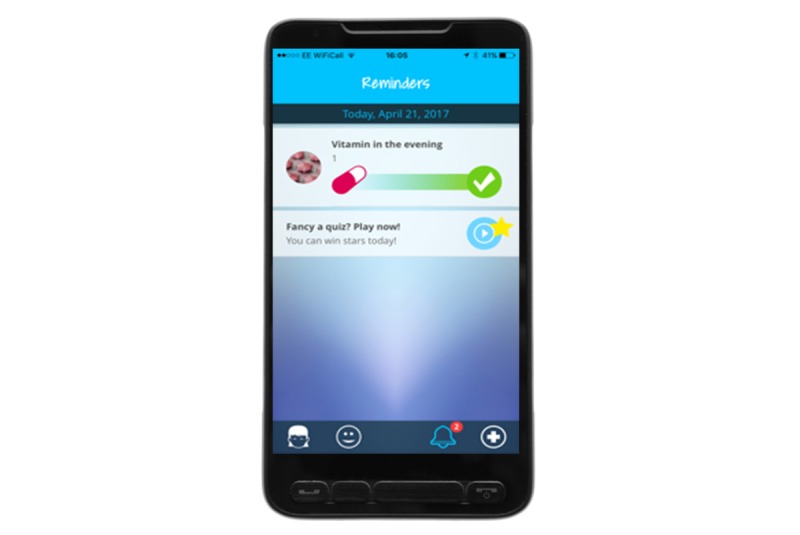
Medication Reminder.

**Figure 7:**
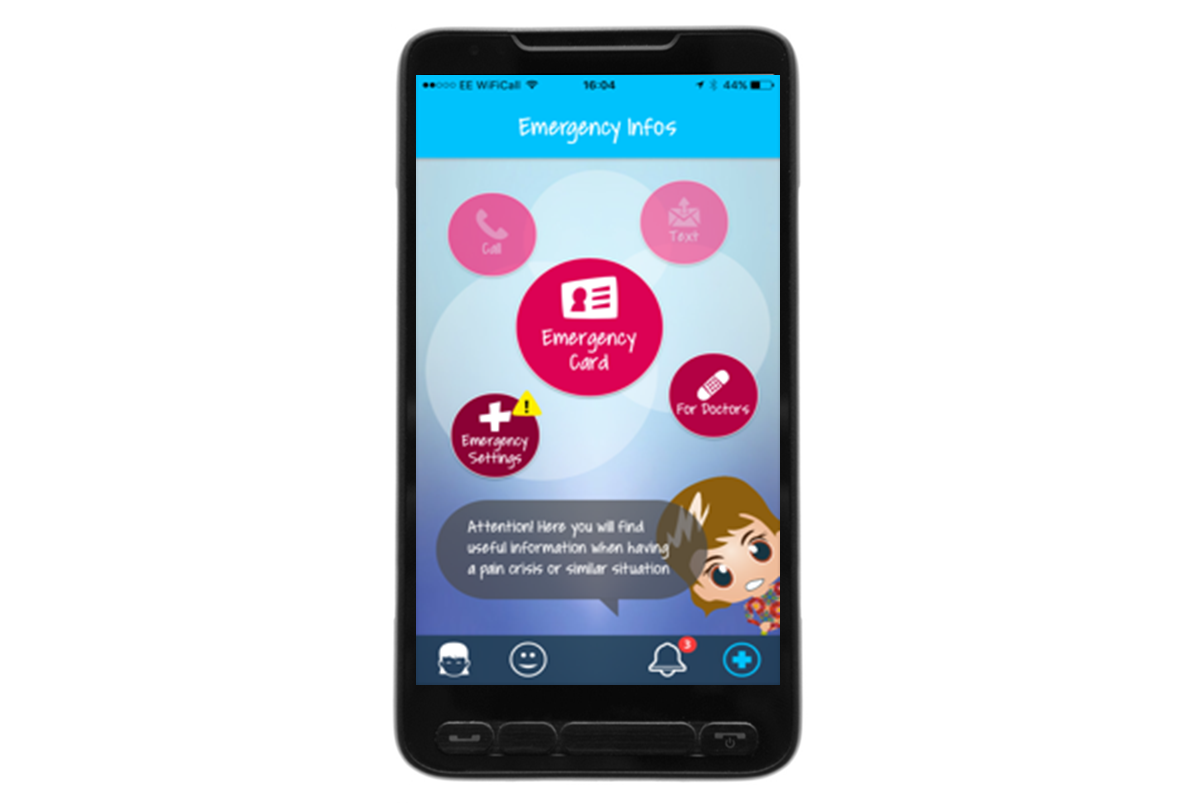
Emergency card.

The following app features were chosen for the first release: *Avatar* (Figures 1 and 2)—a cartoon figure of a boy or girl accompanies the patient throughout his or her interaction with the app. The patient is encouraged to earn bonus points through being active in various app sections to dress the avatar with new clothes, accessories, or even facial hair. *Tip of the day* (Figure 3)—important information on day-to-day coping with the disease, sometimes providing gender-specific tips and dispelling myths. *Daily Quiz* (Figure 4)—points can be earned for answering quiz questions correctly. If a quiz question is answered incorrectly, the right answer is provided, increasing the patient’s awareness of different aspects of his or her disease. *Mood Tracker* (Figure 5)—Users can tilt the phone to indicate which mood they are in. *Medication and Appointment Reminders* (Figure 6)—points are earned for confirming that medication has been taken and appointments have been kept. Patients can see the avatars of other patients using the app and compare their scores. *Emergency section* (Figure 7)—includes crucial information for health professionals unfamiliar with the disease and quick emergency call and text options.

## Discussion

### Principal Findings

Previous research in relation to medication adherence supports the findings in this study, indicating that nonadherence can result from forgetting [40], children disliking the taste [41], and the transition from childhood to adulthood [35]. Chronic illnesses affect young people in a myriad of different ways as they transition into adulthood and adult care [8]. Parents have been largely responsible for ensuring their children self-manage a chronic condition such as taking their medication [8]. Furthermore, evidence suggests that parents’ perceptions of whether the medication will work also predicts medication continuity which aligns with the religious influence on medication adherence reported by one of the participants [42]. Previous research suggests that parents are concerned with their child’s transition into adulthood where there is a need for children to take more control and responsibility toward their condition and to self-manage [33]. Patients need to be able to self-monitor their medication adherence and self-manage their condition as they grow older, and this can supported in the MyMate&Me app through features that enable reminders, self-monitoring, feedback on their behavior, support from peers, and problem solving.

In line with previous research, some participants reported that their mood, stress, and anxiety exacerbated their pain symptoms as reported in previous research with this population [13]. This research also provides new insights among older children where they reported negative emotions around their reliance on medication and its interference with social activities. Furthermore, being outside of the home environment was also cited as a barrier to medication adherence: there were no environmental cues to remind them to take their medication and/or a lack of suitable places to take them in privacy.

Theoretical domains targeted for change were mapped onto 15 BCTs which also align with the components for self-management proposed in the Practical Reviews In Self-Management Support (PRISMS) taxonomy [43]. Although many of these BCTs have been found collectively across medication adherence apps on the market, individual apps have only been underpinned with 2 to 3 of these BCTs. In addition, most have only used Action planning and a Prompt/cue. This study also highlighted the need for BCTs: Goal setting (outcome), Problem solving, and Reducing negative emotions, which are missing in the review of medication apps [22].

### Strengths of the Research

Drawing on a theoretically grounded and evidenced-based intervention development framework, along with conducting research with the target audience, has resulted in the first theory-and-evidence-based app for medication adherence for children and adolescents with SCD. The findings have highlighted barriers that go beyond simply forgetting to take their medication (as focused on in current SDC medication adherence apps), where emotions and social life are perceived to play a pivotal role in medication adherence, particularly during the transition from childhood to adulthood.

### Limitations of the Research

The empirical research engaged a small purposive sample. Consequently, the identified views on the facilitators and barriers to medication adherence may be less representative of other young people with SCD. However, qualitative research may not require a high number of participants before reaching data saturation, and randomization is more suited to quantitative inquiry [44]. The qualitative approach followed, enabled a richer reflection of the barriers and facilitators to the target behavior, and assessed the contribution of the sociocognitive and external factors influencing the target behavior. In addition, the age range of participants may be considered as too wide, raising concerns about the study’s ability to capture the different issues relating to medication adherence. However, the qualitative nature helps to overcome this by allowing participants to give an in-depth account of their experiences.

There are also a number of limitations with the application of the BCW approach. Selecting which BCTs to use in the intervention represented a challenging process as the BCT (v1) at the time of research did not link individual BCTs to their theoretical determinants. However, recently, a Web-based tool has been launched to support this process and specifically links BCTs with theoretical determinants known as *mechanisms of action* [45]. However, as noted by Orji and Mandryk, using a mapping process for intervention development, is always subject to interpretation [46]. The process of selecting IFs was challenging because many of the same BCTs belonged to different IFs. However, the IM table was reviewed by 2 health psychologists to help overcome these challenges.

### Future Research

The app is now undergoing formal usability testing with patients. In addition, nonintrusive data collection such as usage data of app features (which correspond with BCTs) and their correlation to behavior change will help to measure engagement with the intervention as proposed in other mHealth research [47].

### Conclusions

Patients with SCD have complex barriers to medication adherence which can only be identified in using comprehensive enough models and frameworks of human behavior such as the COM-B and TDF. As such, existing apps lacking a theoretical underpinning do not go far enough in supporting young people with SCD; focusing only on 1 or 2 aspects of medication adherence such as reminders and medication logs.

## Abbreviations

BCT: behavior change technique
BCW: behavior change wheel
COM-B: Capability Opportunity, Motivation-Behaviour
IF: intervention function
mHealth: mobile health
SCD: sickle cell disease
TDF: Theoretical Domains Framework
